# Recent Advances in the Use of Cubosomes as Drug Carriers with Special Emphasis on Topical Applications

**DOI:** 10.1155/2024/2683466

**Published:** 2024-07-10

**Authors:** A. Gowri Nath, Prashant Dubey, Ankaj Kumar, Klaudi K. Vaiphei, Jessica M. Rosenholm, Kuldeep K. Bansal, Arvind Gulbake

**Affiliations:** ^1^ Department of Pharmaceutics National Institute of Pharmaceutical Education and Research, Guwahati, Assam 781101, India; ^2^ Pharmaceutical Sciences Laboratory Faculty of Science and Engineering Åbo Akademi University, Turku 20520, Finland

## Abstract

Topical drug delivery employing drug nanocarriers has shown prominent results in treating topical ailments, especially those confined to the skin and eyes. Conventional topical formulations persist with drug and disease-related challenges during treatment. Various nanotechnology-driven approaches have been adopted to mitigate the issues associated with conventional formulations. Among these, cubosomes have shown potential applications owing to their liquid crystalline structure, which aids in bioadhesion, retention, sustained release, and loading hydrophilic and hydrophobic moieties. The phase transition behavior of glyceryl monooleate, the concentration of stabilizers, and critical packing parameters are crucial parameters that affect the formation of cubosomes. Microfluidics-based approaches constitute a recent advance in technologies for generating stable cubosomes. This review covers the recent topical applications of cubosomes for treating skin (psoriasis, skin cancer, cutaneous candidiasis, acne, and alopecia) and eye (fungal keratitis, glaucoma, conjunctivitis, and uveitis) diseases. The article summarizes the manufacturing and biological challenges (skin and ocular barriers) that must be considered and encountered for successful clinical outcomes. The patented products are successful examples of technological advancements within cosmeceuticals that support various topical applications with cubosomes in the pharmaceutical field.

## 1. Introduction

Topical drug delivery systems (TDDS) have been widely employed for treating and managing various skin and ocular diseases. TDDS offers convenience in applying the drug directly to the illness site, patient compliance, localized-targeted drug delivery, and effectiveness at lower concentrations [1]. This has prompted substantial research on TDDS in the pharmaceutical and cosmetic industries. Over the past few years, TDDS has been shown to have various prominent applications in skin and ocular diseases. More than 90% of the ophthalmic products available in the global market today fall within the topical drug delivery category [2]. The WHO reports that approximately 76 million people are affected by glaucoma, 1.5 million by fungal keratitis, and nearly 40% of people suffer from allergic conjunctivitis [3–6]. In addition, topical drug delivery has been extensively explored in treating and managing skin diseases. The data disclosed by the Global Burden of Disease (GBD) in 2019 shows that around 42 million people suffer from skin and subcutaneous diseases worldwide [7]. This diminishes patients' quality of life in terms of social interaction, pain, discomfort, and inability to perform physical activities efficiently [8]. Several factors are responsible for the etiology of skin disorders (psoriasis, alopecia, vitiligo, candidiasis, acne, glaucoma, etc.). These include environmental factors (sun exposure, humidity, pollution, temperature, and radiation), hormonal changes, lifestyle (diet, smoking, and alcohol), stress, trauma, microorganisms (bacteria, fungus, and viral), medications, and cosmetics [9–17]. Genetic factors also play a crucial role in the etiology of skin disorders [18, 19].

**Figure 1:**
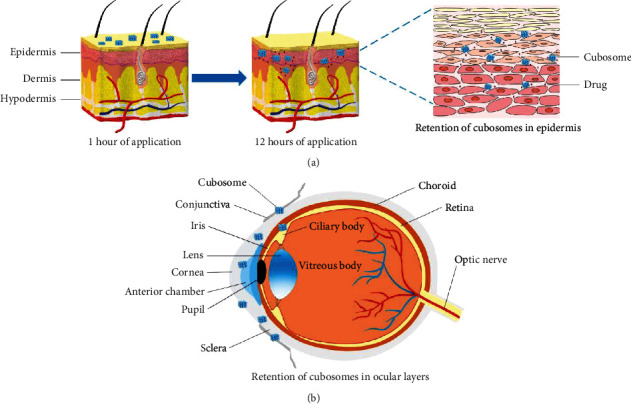
(a) Structure of the skin and CBs (blue) retention in the superficial layers of the skin; (b) structure of the eye and CBs (blue) retention in the ocular layers. Redrawn from [28–30].

Conventional TDDS includes micronized therapeutic embedded creams, gels, ointments, pastes, suspensions, emulsions, powders, and lotions. The management of skin and ocular disease conditions using conventional topical drug delivery is associated with certain challenges, including retention, irritation, allergic reactions, drug-induced hypersensitivity, high drug doses, frequent dosing, reduced penetration, diminished therapeutic efficacy, and increased metabolism with regionally distributed CYP450 enzymes when applied topically. Moreover, conventional formulations account for limited ocular applications due to diverse defense systems and multiple drug entrance obstacles, including rapid nasolacrimal drainage, precorneal drug loss, tear production, and reflex blinking [20, 21]. A drug carrier based on innovative formulations and methods can reduce such issues. Depending on the route of administration, nanotechnology-based drug carriers with sizes ranging from 50 to 500 nm are accepted for drug delivery as nanoparticles. These include liposomes, nanocapsules, cubosomes, dendrimers, niosomes, nanocrystals, solid lipid nanoparticles, and polymeric nanocarriers [22]. Because of their nanosized range, they are endowed with a high surface area, precisely controlled particle size, and low polydispersity to modulate the surface modification, and they can provide better solubility to incorporated drugs, enhanced bioavailability, better penetration through biological barriers, and higher drug retention at the target site for a longer period. Furthermore, they are advantageous in terms of biocompatibility, controlled and sustained drug release, and feasible scale-up technology [22, 23]. Despite the promising future of these nanocarriers, their clinical use is constrained by several issues, i.e., difficulties of drug delivery to the eye's inner structure through local infusion, stability challenges, sterilization issues, high cost, and significant excipient-induced irritation [24]. Given the aforementioned issues, cubosomes (CBs) have shown prominent results in achieving wide applications for topical drug delivery. CBs are liquid crystalline structures that are well retained in the skin and eye regions, providing a suitable environment for loading hydrophobic, hydrophilic, or both types of drugs, exhibiting bioadhesion, and sustaining drug release to achieve a maximum therapeutic effect in topical treatments [25, 26]. The structural resemblance between the cubic phase of CBs and the skin's *stratum corneum* aids in achieving more efficient CBs penetration through this outermost layer of the skin epidermis [27]. The biomembrane-typical framework of the cubic phase improves physical adherence to the skin, thus enhancing the retention of CBs in different layers of such organs (Figure 1) [28–30]. In addition, the ease of formulation, high drug-loading ability, greater stability at any dilution level, great resistance to breakage, low toxicity, and economical nature of CBs make them ideal drug carriers for topical application [31]. This led to a widening of research on nonlamellar reversed lyotropic liquid crystals dispersed in aqueous media, including hexagonal mesophases, micellar discontinuous cubic phases, and bicontinuous cubic mesophases [32].

This review explains the role of the topical applications of CBs for skin disorders and eye diseases. This study further highlights the challenges associated with conventional drug carriers in topical applications and how these can be solved using CBs. The topics covered include the development of CBs, their components, methods of preparation and characterization, factors affecting CB formation, release kinetics, and recent advances. The wide variety of applications, patents, and clinical trials related to the promising role of CBs against skin disorders and eye diseases are discussed in the review.

## 2. Conventional Treatment Options for Topical Diseases, Their Challenges, and Possible Solutions

Conventional treatment options and their challenges are discussed in this section. Various marketed products are useful for skin and ocular conditions; however, they are generally associated with certain challenges, as shown in Table 1, that need to be considered to gain effective market potential.

The ideal topical drug carrier possesses high encapsulation efficiency and monodisperse particles with small particle size, retention, and stability under physiological and storage conditions. Conventional liposomal formulations are limited to topical applications because of chemical and physical stability challenges. Chemical stability includes oxidation and hydrolysis of the phospholipids, whereas physical instability may be governed by the low surface charge of liposomes, thus hampering structural stability [33, 34]. The lower encapsulation efficiency of hydrophilic drugs and their delivery to deeper skin layers are other challenges that restrict the topical applications of conventional liposomes. Technological advances have driven various liposomal formulations that overcome the challenges associated with conventional liposomes [35]. It includes ethosomes, transfersomes, niosomes, vesosomes, and cubosomes. Each vesicular system has its pros and cons, e.g., toxicity challenges are present in niosomes owing to the use of esters, chemical instability, and hydrophobic active constituents alter the elasticity of transferosomes, and low yield and noneconomic effect of ethosomes are some of the challenges associated with them [36, 37]. Compared to liposomes, CBs have a greater membrane surface area for encapsulating more hydrophilic and hydrophobic drugs [38], deeper skin layer permeability [39], better storage stability, and lower leakage of encapsulated drugs [40].

Rattanpak et al. conducted a comparative study using four different types of lipid-based formulations for topical codelivery of monophosphoryl lipid A (MPL) and Quil A (extract of bark of *Quillaja saponaria* tree), i.e., liposomes, transfersomes, ethosomes, and cubosomes. The preparation of liposomes and transfersomes was carried out by a thin film hydration method, ethosomes were formed by the reverse phase method, and CBs were prepared by the lipid precursor method. The particle size, PDI, and ZP of MPL-Quil A-liposomes were found as 164.9 ± 14 nm, 0.22 ± 0.07, and −21.1 ± 7.0 mV; for MPL-QA-transfersomes, 135.9 ± 14 nm PS, 0.17 ± 0.06 PDI, and −21.3 mV ZP; for MPL-QA-ethosomes, 200.5 ± 32 nm PS, 0.21 ± 0.04 PDI, and −14.2 ± 4.0 mV ZP; and CBs possesses 183.7 ± 6 nm PS, 0.17 ± 0.03 PDI, and −27.5 ± 1.0 mV ZP. An *in vitro* skin permeation study was conducted on the skin of still-born piglets. The enhanced permeation of the peptide was reported from CBs compared with liposomes, ethosomes, and transfersomes, thereby depicting CBs as an effective topical formulation [39].

## 3. Cubosomes and Their Components

**Figure 2:**
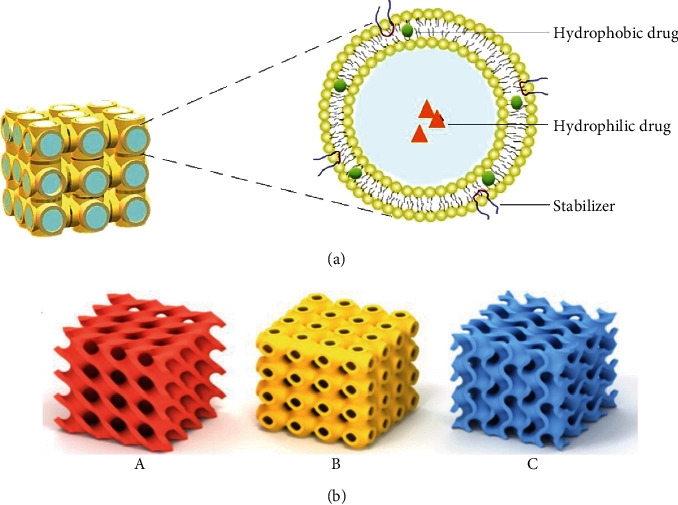
(a) Cubosomal structure; (b) 3D schematic representations of bicontinuous cubic phases: A *Ia3d* (gyroid type, CG), B *Pn3m* (double-diamond type, CD), and C *Im3m* (primitive type, CP). Reproduced with permission [61]. Copyright © 2022 ACS Publications.

CBs are nanosized liquid crystalline, inverse bicontinuous lipid layers, and three-dimensional (3D) structures consisting of crystallographic symmetry, formed by the self-assembly of amphiphilic lipids in polar solvents without intersecting the aqueous channels to form a thermodynamically stable structure [53, 54]. The general structure of the CBs is illustrated in Figure 2(a). Based on the composition of lipids and additives/stabilizers, the lipid layer in the bicontinuous cubic phase could have a thickness of ∼3.5 nm and a water channel diameter of 3-25 nm that can vary significantly while the interfacial area can be up to ∼400 m^2^ g^−1^, thus being able to offer a sophisticated diffusion channel for prolonged release of entrapped drugs [55, 56]. Lyotropic liquid crystals can self-assemble into a variety of geometries, such as the inverse bicontinuous cubic phase (CBs) (QII), hexagonal phase (HII), micellar cubic (III), and emulsified microemulsion (LII) [57]. Of these geometries, CBs have special features due to their unique structure and a greater interfacial area between the lipid and water.

The liquid crystalline structures of CBs provide a suitable environment for high loading of drugs (hydrophobic or hydrophilic or both types), exhibit bioadhesion, have controlled and delayed release performance, and help to reach inside the regions of the eye and skin [14]. Before approaching these applications, a basic understanding of CBs, their types, components, and methods of characterization is required. Generally, CBs are divided into two categories based on their nanostructure, i.e., the bicontinuous phase or the discontinuous phase, both of which consist of water compartments. Furthermore, the bicontinuous cubic phase can be further classified on the basis of the variations in nodal surfaces, i.e., *Ia3d* (gyroid type, CG), *Pn3m* (double-diamond type, CD), and *Im3m* (primitive type, CP) assemblies, as shown in Figure 2(b) (A–C). On the other hand, the symmetry type *Fd3m* (discontinuous type) is mostly present in a heterogeneous system of the discontinuous cubic phase, which comprises two distinct quasispherical close-packed micelles in a 3D cubic lattice [58]. A reduction in the mean curvature occurs from *Ia3d*, *Pn3m*, and *Im3m*, but an increase in the water-retaining capacity occurs from *Im3m* to *Ia3d* [59]. The CBs in the bulk phase possess a *Pn3m* nodal surface. Upon homogenization, the cubosomal structure of the *Pn3m* assembly is transformed to *Im3m* [60].

### 3.1. Components of CBs

Conventional liposomal formulations are physically and chemically unstable systems [26, 27]. Physical instability arises due to inter- and intraliposomal forces that drive from bending energy and cohesive forces among the hydrocarbon chains. The forces give the system a spherical shape and can lead to the leakage of loaded components. The bending energy of the phospholipid bilayer is also responsible for providing thermodynamically unstable colloidal systems. The lipids used in the liposome formulations are mostly obtained from biological origins, e.g., egg lecithin and soybean lecithin, and comprise polyunsaturated fatty acids, which are highly unstable. The unsaturated lipids are more vulnerable to oxidation, thus diminishing the shelf life of liposomes [62, 63]. The structural components of CBs include lipids (monoolein, oleyl glycerate, and phytantriol), surfactants, and stabilizers. Monoolein is a mixture of glycerides of oleic acid and other fatty acids that can be susceptible to esterase-catalyzed hydrolysis [64]. Thus, the structural instability of monoolein-based CBs limited their practical applications. Phytantriol offers higher structural stability to CBs because of the absence of ester linkage and the presence of a saturated phytanyl backbone. Phytantriol-based stable cubic phases exist in equilibrium with excess water, which is an optimum environment for the formation of CBs. In addition, phase transitions (Q_II_ to H_II_) for the phytantriol/water systems were observed at lower temperatures (40°C). Thus, there is a higher possibility of forming stable HII phases at much lower temperatures [65]. The internal nanostructure of the CBs consists of different aqueous and nonaqueous channels, thus making it feasible to load hydrophilic and hydrophobic moieties. The inner interior surface area of CBs offers a higher loading of biologics and reduces the chances of drug leakage [66].

Other lipids used to prepare CBs include dipalmitoyl phosphatidylserine (DPPS) and monoelaidin. Likewise, Pluronic F108, Pluronic F87, Pluronic F68, Poloxamine 908, etc., are used as stabilizers because of their amphiphilic (surfactant) nature [67]. The hydrophobic part of the lipid that facilitates the partitioning of the hydrocarbon chain in water leads to the spontaneous formation of mesophases. In contrast, the hydrophobic chains are partitioned to limit oil–water interaction, and the hydrophilic group is preferentially hydrated at the interface of water. Moreover, incorporating stabilizers in CBs also affects the formation of mesophase, toxicity, or protection from hydrolysis [68].  Table 2 describes the various types of lipids and stabilizers employed for the successful engineering of CBs.

## 4. Factors Affecting the Structure of CBs

### 4.1. Concentration of Stabilizer or Surfactants

The concentration of stabilizer employed in a cubosomal formulation plays a crucial role in the type of CBs obtained. Smaller particles with good stability were formed when the concentration of the stabilizer was high (1-1.6%) because of faster hydration of the stabilizer's head groups. Hydration causes stabilizer incorporation into the cubosomal structure and, thus, stabilizes the cubic structure. However, if the stabilizer concentration is less than 1%, the high-energy dispersion process leads to the formation of less stable particles due to low hydration and partial adsorption of the stabilizer [76]. The most commonly used stabilizers for cubosomes are steric stabilizers e.g., Pluronic triblock copolymer F127. Various studies have investigated each stabilizer's underlying mechanism and concentration-dependent effect on CBs formation. The stabilizers affect the particle size, PDI, and %EE of the CBs, as investigated by Rapalli et al. The authors determined that stabilizer concentrations positively influenced the %EE of the hydrophobic drugs [53]. However, the particle size and PDI of the system solely depend on the nature of the stabilizers. A study by Chong et al. investigated novel steric stabilizers for CBs. The authors assessed the Pluronic F127, Cremophor EL, Tween 80, Phytosterols (BPS-05 and 30), D-*α*-tocopheryl poly (ethylene oxide) 1000 succinate (TPGS), Myrj 10, 20, 25, 40, 45, 52, 55, 59, and PEO 100 units, and poly (3-hydroxybutyric acid-co-3-hydroxyvaleric acid) as stabilizers with different concentrations. The CBs were prepared using phytantriol and monoolein with four different concentrations of each of the 16 stabilizers (0.1% wt, 0.5% wt, 1.0% wt, and 2.0% wt). Pluronic F127 and Myrj 59 were effective with phytantriol to produce uniform milky dispersions among all stabilizers. However, Myrj 59 was effective at a lower concentration of 0.1% wt in contrast to the 0.5% wt concentration of pluronic F 127. The particle size and PDI observed with Myrj 59 were 380.7 nm and 0.46, respectively, whereas smaller uniform particles were obtained when Pluronic F127 was used as a stabilizer (PS 193.0 nm and PDI 0.16). It was also reported that the stabilizer influences the internal structure of CBs. Thus, an investigation was conducted using synchrotron SAXS. Among the abovelisted stabilizers, Myrj 59 was further assessed owing to the improved stability of the system. The Bragg reflections were obtained at different positions of 2^1/2^, 3^1/2^, 4^1/2^, 6^1/2^, and 8^1/2^, which indicated that the double diamond phase was retained for the cubic phase formed from phytantriol despite the change in concentrations of Myrj 59. However, different results were obtained for CBs prepared from GMO with Myrj 59 and Pluronic F127. At a concentration of 0.5% wt, 7 Å greater lattice parameters were obtained than those of Myrj 59 (153 Å). Finally, it was concluded that Myrj 59, with a longer PEO chain, offers higher hydrophobicity than Pluronic F127, which can stabilize the CBs formed from phytantriol at lower concentrations [77]. Technological advances have driven various other classes of stabilizers for the biological and structural stability of CBs that offer higher internalization and fusogenicity. Examples are poly (N, N-dimethylacrylamide)-block-poly (N-isopropylacrylamide (PDMA-b-PNIPAM) [78] and poly (methyl acrylate) (PMA) and poly (poly (ethylene glycol) methyl ether acrylate) (PPEGMA) [79, 80].

Various types of surfactants are used to form different liquid crystalline structures. The type and concentration of surfactants determine the group spacing among the liquid crystalline phases. Surfactants are well known to reduce the surface tension between the two-phase system, usually the oil and water phase in cubosomes. As per various studies, increased concentration of surfactant reduces the particle size and PDI of the CBs owing to a reduction in surface tension. In addition, a positive correlation was reported between the concentration of surfactant and the percentage entrapment efficiency of hydrophobic and hydrophilic moieties [53, 81, 82]. Yakaew et al. investigated the effect of various concentrations of poloxamer-188 (Pluronic 68 or F68) on CBs. The low-energy stirring method was used to produce F68-based CBs following a small-angle X-ray scattering study to determine the interlayer spacing of the LC phases. The increase in concentration over 40% yields a viscous gel, followed by a hard gel with a further rise in F68 concentration. At a concentration < 40% of F68, no liquid crystalline phases were observed, with the lowest elastic modulus value (G'). The authors reported that an increase in F68 concentration follows the sequence of phases as Im3m cubic-Fd3m-micellar cubic phase and P6mm phase [83].

Another study by Nagao et al. investigated the role of Gemini surfactant (Sodium dilauramidoglutamide lysine, DLGL) in the colloidal and structural stability of CBs. A high-energy process (probe sonication) was used to prepare phytantriol (PHY) and DLGL-based CBs. Different ratios of DLGL/PHY (1%-30%) were studied to assess the effect of DLGL concentration on phase structure, PS, and PDI. The PS of the prepared CBs was directly correlated with the DLGL/PHY ratio. However, no correlation was associated with PDI. The internal structure of the samples was determined using SAXS, and no cubic crystalline phases were observed at 0% w/w DLGL/PHY. At concentrations of 1% and 3% w/w, diffraction patterns for the CBs were observed at a spacing ratio of 2^1/2^ : 3^1/2^ : 4^1/2^, and those peaks were governed for cubic space group Pn3m. A further increase in concentration > 5% or 6% w/w disrupts the formation of CBs and transforms them into small unilamellar vesicles. The prepared samples were found to be stable at a 2% DLGL/PHY concentration, confirming the adequate concentration to form stable Im3m cubic structures [84].

### 4.2. Phase Transition Behavior of GMO and PHY

**Figure 3:**
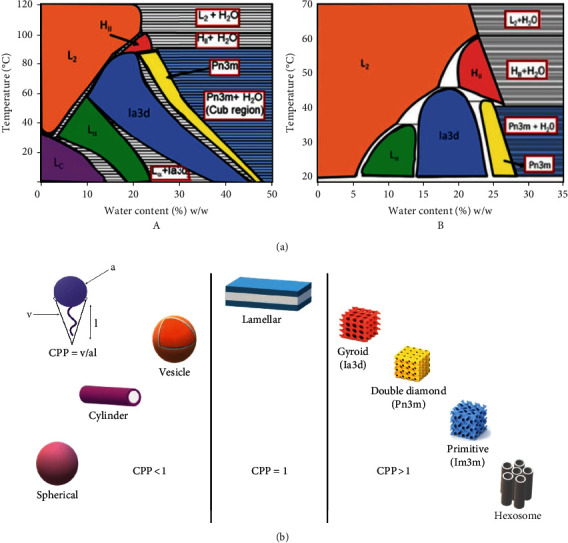
(a) Schematic representation. Phase transition behavior of the A GMO-water system and B PHY-water system with varying temperature and water content. (b) Critical packing parameter (CPP < 1) forms normal shape assemblies (spherical, cylinder, and vesicle), (CPP = 1) forms lamellar, and (CPP > 1) forms reverse self-assemblies (CBs and hexosomes). (a) Reproduced with permission [85]. Copyright © 2022 https://creativecommons.org/licenses/by/4.0/.

Based on the temperature and water content, the GMO-water system existed in different phases (Figure 3(a), A). With increasing temperature and water content, the phase shifts from lamellar to inverted bicontinuous cubic phases (*Ia3d*, followed by *Pn3m*). On a further increase in the water content, the *Pn3m* cubic phase begins to coexist with the extra water. The GMO–water system generates inverted hexagonal and micellar phases at high temperatures with or without the presence of additional water. In contrast, a reversed micellar phase is formed in the PHY-water system at low temperatures and water contents. However, an increase in water content leads to the formation of lamellar to inverted bicontinuous cubic phases (*Ia3d*, followed by *Pn3m*), as previously observed in the GMO–water system. In addition, reversed micellar and hexagonal phases are favored at high temperatures and depend on the water content [85]. A phase transition of the PHY-water system is depicted in Figure 3(a), B.

Rajesh et al. investigated nine synthesized ionizable amino lipids for the phase transition mechanism in CBs. The amino lipids contain a hydrophobic oleyl tail and hydrophilic head group. Lipids 1-4 were classified as pyridinyl oleates, lipids 5 and 6 as heterocyclic oleates, lipid 7 as aniline oleate, and lipids 8 and 9 as dioleates. The high-throughput method was used to prepare amino lipid-based nanoparticles with a particle size of 176-298 nm, 0.09-0.23 PDI, and they were found to be stable for 30 days with no significant change in particle size. It was observed that an increase in amino lipid content at different pH levels changes the mesophase structure from bicontinuous to cubic to hexagonal to inverse micellar. The nanoparticles doped with lipid-1 and lipid-2 phase transitions were observed from the diamond cubic phase to H_2_ (Q_2_^Pn3m^ to H_2_) at a concentration *R*_MO_ = 0.15. Lipid-3 showed a phase transition of Q_2_^Pn3m^ to H_2_ at a lower concentration *R*_MO_ = 0.1, and lipid-4 showed a phase transition of Q_2_^Pn3m^ to H_2_ at *R*_MO_ = 0.3 under neutral conditions. Lipid-5 showed phase transitions from Q_2_^Pn3m^ to (Q_2_ + H_2_) at *R*_MO_ = 0.2, whereas for Q_2_^Pn3m^ to H_2_ at *R*_MO_ = 0.25. A lower concentration *R*_MO_ = 0.1 was determined for lipid-6 for phase transition to H_2_. The higher lipophilicity of the molecule was governed by phase transitions from lipids 7, 8, and 9 at lower concentrations at *R*_MO_ = 0.1 − 0.15. Finally, we concluded that the type of lipid and pH influence the phase transitions in the cubic phase system [86].

### 4.3. Phase Transition in CBs due to the Presence of Guest Molecules

In general, CBs have been well explored as drug carriers to deliver therapeutic entities effectively at the desired site. These therapeutic entities or guest molecules include drugs, proteins, vitamins, and peptides. These lipophilic, hydrophilic, and amphiphilic guest molecules can be prevented by glycerol monooleate-mediated liquid crystalline cubic phases for their degradation (oxidation or hydrolysis) [87]. After investigating lysozyme as the first guest molecule, numerous studies have been conducted to explain the phase transitions of CBs in the presence of guest molecules [88]. The same research group investigated the phase behavior of the cubic phase using proteins, bovine serum albumin, and pepsin. The authors concluded that high net charges in proteins generally influence the creation of cubic phases owing to ionic-ionic interactions. Further investigation was conducted using different amino acids and peptides (lysine, vasopressin, desmopressin, somatostatin, and renin inhibitors) and found that above a certain concentration, there was a change in phase transitions from the cubic phase to the lamellar phase. It was electrostatic repulsion governed through peptides with the glycerol-monooleate interface [89].

In addition, various drug molecules (bupivacaine [90], gentamicin sulfate [91], clonidine [92], cyclosporine [93], etc.) have been investigated for phase behavior and release studies from cubic phases. As per the nature of the guest molecules, the hydrophobic molecules are generally entrapped in the lipid aliphatic regions, whereas hydrophilic molecules exist within the aqueous water channels. However, amphiphilic therapeutics are present at water-lipid interfaces. Hydrophobic guest molecules (diazepam and prednisolone) in the lipid membrane increased the curvature of the lipid layer, thus inducing the phase transition. Hydrophilic drugs, on the other hand, require higher concentrations to achieve a higher payload of CBs [56].

Several key aspects, including drug loading and release, highly depend on the cubic phase's phase behavior when incorporating guest molecules. The thermal phase behavior of the binary PHY-water and ternary PHY-vitamin E acetate- (VitE-A-) water systems was investigated. The lipophilic materials, even in minimal concentrations, might considerably influence the phase behavior, as evident by the fact that lipophilic VitE-A in the PHY system decreased the temperature of the QII to HII to LII transitions. Another comparison was made between the impact of DL-*α*-tocopheryl acetate and other lipids on the phase behavior of the GMO system, including limonene, triolein, tetradecane, and DL-*α*-tocopherol. Gradually adding DL-*α*-tocopheryl acetate to a binary unsaturated monoglyceride mixture caused a reversed cubic phase, a reversed hexagonal phase, and a reversed microemulsion to form when too much water was present. However, the solubilization of some molecules, such as DL-*α*-tocopherol, results in the existence of the reversed micellar cubic phase but not in the solubilization of other molecules. Bicontinuous CBs undergo structural changes with increased oleic acid, leading to hexosomes, micellar cubosomes, and emulsified microemulsions. When oleic acid was present in sufficient concentrations, it was discovered that the internal structure of CBs also strongly depends on the pH of the aqueous phase. As the pH increases, transformations can be observed from emulsified microemulsions to micellar cubosomes, hexosomes, and bicontinuous cubosomes to vesicles. [69]

## 5. Effect of the Type and Concentration of Lipids

Generally, amphiphilic lipids consisting of partially hydrophilic moieties known as the head and hydrophobic moieties known as the tail form cubic phases. Lipid structure and interactions in the aqueous environment drive their phases. The formation of CBs occurs when a lipid mixture, along with a stabilizer and molecule of interest, self-assembles to form a lipid bicontinuous cubic phase [32]. In the cubic phase structure, the competition among the free energy, i.e., curvature energy and stretching energy, is the basis of the spontaneous formation of CBs and their thermodynamic stability. A higher water level causes swelling that transforms the G-surface into the D-surface of the GMO-water system. The P-surface can be formed only by adding a third component, i.e., an amphiphilic block copolymer [69].

The different morphologies of lipids and their self-assembled frameworks that occur due to solvent influence can be understood by the critical packing parameter [32]. The critical packing parameter (CPP) gives an idea of the geometry of the structure. It is defined as CPP = *v*/*al*, where “*v*” and “*l*” are the volume and length of the hydrophobic chain, respectively, and “*a*” is the surface area of the hydrophilic group at the head [94]. The self-assembled structure and its structural transformation in amphiphilic solutions can be explained and predicted using CPP, which is a potent tool. However, until now, rather than being empirical, the assessment of such a crucial quantity has been considered speculative. The concentration of molecules and other components in the system influences the CP*P* value [95].

In addition to *Ia3d*, *Pn3m*, and *Im3m*, different shapes (spherical, cylindrical, vesicle, lamellar, and hexosomes) are also governed by the CPP values, as illustrated in Figure 3(b). If CPP is <1, spherical, cylindrical, or vesicle-shaped particles are formed. If CPP = 1, the formation of lamellar-shaped particles is usually observed. However, if CPP > 1, the formation of *Ia3d* (gyroid), *Im3m* (primitive), and *Pn3m* (double-diamond) types of CBs as well as hexosomes occurs [96]. The increase in negative curvature tends to transition from the cubic phase to hexoses or emulsified microemulsions by the addition of hydrophobic constituents such as oils. The reversal of negative curvature can be achieved by adding a surfactant to form the previous cubic phase [97]. Many parameters, such as pressure, ionic strength, pH, and temperature, influence the CPP of a lipid bilayer. It is believed that the CPP of the amphiphiles alters the bilayer geometry [61].

## 6. Mechanism of Drug Release from CBs

The small pore sizes of CBs facilitate tortuous diffusion to achieve controlled release of drugs from the regular channel of the ribosomal structure. Entrapped drug release is mostly dependent on molecular weight and polarity. Hydrophilic drugs have immediate drug release because of an increase in the contact surface area with the aqueous environment. In contrast, the release rate of hydrophobic drugs is curtailed owing to the strong affinity of drugs for the cubic phase's hydrophobic region [68]. According to the mathematical model, amphiphilic drugs align at the lipid–water interface and exhibit complicated release behavior. This behavior may be due to the partition coefficient, drug diffusion in the water channels, and drug diffusion in the lipid bilayer. The release of drugs from CBs generally follows the Higuchi-diffusion-controlled kinetic law (Equation (1)) or the Korsmeyer–Peppas equation (Equation (2)) [98].

(1) $$\begin{array}{c} Q={\left[Dm\;Cd\;\left(2A-Cd\right)t\right]}^{1/2}. \end{array}$$

The equation states that the square root of time affects how agents are released from the matrix, where *Q* is the quantity of agents released per unit area of the matrix, *Dm* is the diffusion coefficient of the agent, *Cd* is the solubility of agents in the matrix, *A* is the concentration of agent present in the matrix per unit volume, and *t* is the time.

(2) $$\begin{array}{c} F=\left[\frac{M_{t}}{M}\right]=K_{m}\;t^{n}, \end{array}$$

where *F* is the fraction of drug released at time *t*, *M*_*t*_ is the amount of drug released at time *t*, *M* is the total amount of drug in dosage form, *K*_*m*_ is the kinetic constant, *n* is the diffusion or release exponent, and *t* is the time in hours.

Makhlouf et al. explained the release kinetics models of the prepared minoxidil CBs (MXD-CBs). The CBs exhibited 131.10 ± 1.41 nm particle size, −23.5 ± 0.42 mV zeta potential, with a polydispersity index of 0.18. The percentage encapsulation efficiency for MXD was found to be 80.4 ± 4.04%. The MXD release curve fits well with the Higuchi diffusion kinetics on the basis of the higher regression coefficient value (*R*^2^). The exponent “*n*” in the Korsmeyer–Peppas equation was calculated to be 0.6792, indicating non-Fickian or anomalous drug release (i.e., diffusion and erosion-controlled release) [99].

## 7. Surface Modification of CBs

Recent advances in technology-driven chemical surface modification approaches for CBs have imparted the attachment of polymers, aptamers, proteins, or peptides to the surface of CBs. Modification can be performed on CBs to meet the various requirements in different biomedical applications as a core lipid matrix made up of lipids and additives with adjustable nanostructure, functionality, or responsiveness that can be used for smart drug delivery [56]. The surface of CBs can be modified to improve their physical and biological stability by coating them with polyethylene glycol (PEG) or chitosan. For instance, the loss of the mucoadhesive capability of CBs due to water absorption necessitates surface coating with polyelectrolyte material to improve mucoadhesiveness [100]. Direct mixing of positively charged polyelectrolytes and negatively charged CBs results in the single-layered coating of CBs. Chitosan (CH) is one of the most widely used coating materials owing to its cationic and mucoadhesive nature [101]. Said et al. explained the usefulness of chitosan-based coating on CBs surfaces to improve their mucoadhesive properties. Chitosan-coated CBs (CH-CBs) were prepared by direct addition of CH to the CBs formulation using 1% acetic acid as a diluent to solubilize CH. It was observed that CH-CBs interacted with mucin more significantly than neat CBs formulations with an increase in particle size (PS) and zeta potential (ZP) (CH-CBs (PS: 2449 ± 155 nm, ZP: 39.10 ± 0.22 mV)) (CBs (PS: 102 ± 0.07 and ZP: −9.20 ± 0.35 mV)). The results revealed that excellent surface modification was achieved because of the ionic interaction between CH and CBs. The overall findings from the study explained the importance of CH as a coating agent to enhance the efficacy of topical ocular antifungal treatment and that it can also be applied to other topical applications [102]. The surface modification approach of CBs may also be prominent toward the deliverability and targetability of bioactive agents, as investigated in various articles [103, 104]. However, more studies relevant to this need to be explored in the future to meet advanced topical applications.

## 8. Method of Preparation and Characterization of the CBs

### 8.1. Method of Preparation

**Figure 4:**
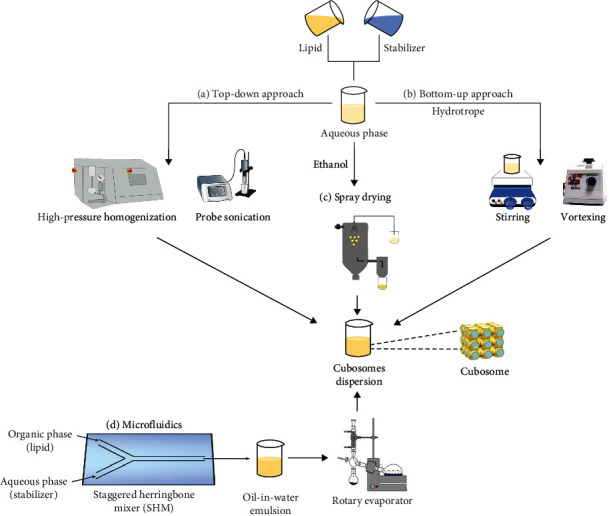
Method of preparation of CBs using different approaches. Adopted and redrawn from [107–110, 113–115].

The method of preparation of CBs consists of many techniques, of which commonly used approaches such as top-down, bottom-up, spray drying, and microfluidics are discussed below. The steps involved in these approaches are shown in Figure 4. In addition, the advantages and disadvantages of these approaches are discussed in Table 3. Moreover, examples of CBs produced using different manufacturing techniques are listed in Table 4.

Usually, three macroscopic types of cubic phases are noticed during preparation: precursor form, bulk gel form, and particulate dispersion form. The precursor form is an existing solid or liquid substance that transforms into a cubic phase upon contact with a liquid. The solid-like bulk cubic phase gel in equilibrium with water forms CBs particles [105]. As previously mentioned, various deciding factors, such as the concentration of lipids, stabilizers, and process variables, influence the formation of CBs particles. Loading therapeutic agents in CBs involves localization of the drug within the water channels in the cubic phase, loading within the lipid bilayer, or adhering to the lipid membrane. The therapeutic agent may either be mixed with the molten lipid or lyophilized along with the lipid film before being dispersed to achieve loading of the drug moieties. Conversely, the incubation process is another method that can be used to load therapeutic moieties onto CBs dispersions that have already been formed [60].

#### 8.1.1. Top-Down Approach

The most commonly used approach in the preparation of CBs is the top-down approach. Briefly, the lipid is added to water premixed with a stabilizer, and the bulk phase of the viscous liquid crystal is formed [66]. Subsequently, a stable formulation is formed using high-energy dispersion techniques such as high-pressure homogenization or high-shear homogenization with probe sonication. The top-down approach also includes the hot emulsification method, which involves preheating the lipid phase over the glass transition temperature and, subsequently, adding it to the heated aqueous phase to make the primary emulsified system. The emulsified system was further processed using high-energy processes to reduce the particle size of the carrier [106]. Figure 4(a) illustrates the brief methodology of the top-down approach [107].

#### 8.1.2. Bottom-Up Approach

This is referred to as the solvent dilution or liquid precursor method. It usually entails dispersing a lipid-forming liquid crystal, a polymer, and a hydrotrope in excess water to form separate nanoparticles with less energy input, such as sonication, vortexing, and stirring, as shown in Figure 4(b) [108–110]. Normally, a hydrotrope (ethanol) is used for hydrophobic lipids in aqueous media for their solubilization and long-term stability of CBs formation [111].

#### 8.1.3. Spray-Drying Approach

In this method, a powder form of CBs is described using organic solvents that evaporate when exposed to air. A wave of hot air was used to atomize the lipid-surfactant solvent mixture, which caused the solvent to evaporate rapidly and led to the formation of a dry powder of CBs. Initially, the lipid and stabilizer mixture were dissolved in ethanol (binary solutions are also used instead of ethanol). A separate aqueous phase mixture consisting of a hydrophilic solid carrier, such as sorbitol or dextran, is then mixed with the initially prepared lipid mixture [112]. Figure 4(c) depicts the steps involved in the spray drying method [113].

#### 8.1.4. Microfluidics Approach

Microfluidics is an advanced technique for synthesizing CBs. Specifically, the SHM (staggered herringbone mixer) device (Figure 4(d)) is used to fabricate CBs. The device consists of two inlets, where the lipid dissolved in ethanol is loaded in the first inlet and the aqueous phase is loaded in the second inlet using a syringe pump. The total flow rate and the flow rate ratio of the lipid and aqueous phases must be optimized to produce the defined size of CBs. The microchannels of the herringbone structure induce chaotic advection in the ethanol and aqueous solutions, resulting in intermediate Reynolds numbers (2 < Re < 500 for 0.02 ml/min < total flow rate < 4 ml/min). After attaining these conditions, a nanoemulsion is formed with a lipid layer stabilizing the two solvents' interface. The formed nanoemulsion is collected from the outlet and then subjected to rotary evaporation for solvent (ethanol) evaporation. Finally, a CBs dispersion with a small particle size and narrow size distribution is obtained [114, 115].

### 8.2. Characterization of the CBs

The primary characterization of CBs includes hydrodynamic particle size, PDI, and zeta potential measurements. The surface and internal morphologies of the particles can be identified by scanning electron microscopy and cryo-transmission electron microscopy, respectively. The identification of phases is usually determined by small-angle X-ray scattering, differential scanning calorimetry, and X-ray diffraction, which are employed to understand the phase transition and crystallinity. The different techniques used in the characterization of CBs are listed in Table 5.

## 9. Applications of CBs in Various Skin Diseases

The applications of CBs are abundant due to their liquid crystalline structure, making them appropriate for delivery through mucosal and topical routes. The bioadhesive nature of CBs efficiently distributes to the surface and inner layers of skin in an effective way. The research community has explored CBs for various skin diseases such as psoriasis, skin cancer, cutaneous candidiasis, acne, alopecia, and vitiligo, adopting strategies that will be described in the following.

### 9.1. Psoriasis

Psoriasis is a chronic inflammatory skin condition with significant hereditary propensity and autoimmune pathologic characteristics. Globally, 2% of people suffer from psoriasis with several regional differences [135]. It has complex pathogenesis, including changes in the skin's immune system, epidermal hyperproliferation, and inflammation. Higher DNA synthesis and a noticeably lower rate of epidermal turnover are two features of hyperproliferation. An influx of T-lymphocytes in the dermis, with a predominance of CD8+ cells, and infiltration of neutrophils in the epidermis and superficial dermis cause inflammation [136, 137]. The beneficial properties of CBs, like nanometer size range, greater permeation capacity, and better retention for longer contact time with skin, make them prominent drug carriers for treating psoriasis [138].

**Figure 5:**
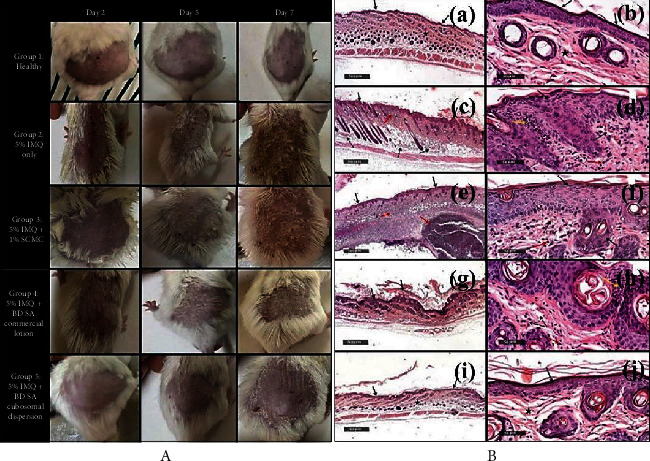
(A) The effect of different formulations of different days (2, 5, and 7) on psoriasis management. (B) The micrograph of X-500 *μ*m and X-50 *μ*m magnified dorsal skin of balb/c mice: a, b healthy group, c, d received 5% IMQ, respectively, e, f received 5% IMQ+blank CBs in 1% SCMC, respectively, g, h received 5% IMQ+BD-SA marketed lotion, respectively, and i, j received 5% IMQ+BD-SA-CBs. Reproduced with permission [138]. Copyright © 2022, Dove Medical Press Ltd.

For the management of scalp psoriasis, Shalaby et al. prepared betamethasone (BD) and salicylic acid (SA) coloaded CBs dispersion using the top-down approach. The optimized particle size and PDI of BD-SA-CBs were found to be 197.4 ± 9.745 nm and 0.443 ± 0.025, respectively. They performed the *in vivo* study on male balb/c mice for 2, 5, and 7 days where the animals were divided into five groups, *viz*., healthy group, 5% imiquimod (IMQ) group, 5% IMQ + 1% sodium carboxymethylcellulose (SCMC), 5% IMQ + BD-SA commercial lotion, and 5% IMQ + BD-SA-CBs. The psoriasis area and severity index (PASI) scoring were given to determine the severity of inflammation and psoriasis in IMQ psoriasis-induced mice. As depicted in Figure 5(A), it was observed that BD-SA-CBs showed superior action toward a decrease in reddish skin and scaly lesions over the other groups, further recovering rapidly with highly ordered histological arrangements of the skin. With different magnifications of skin micrographs, as shown in Figure 5(B), a huge reduction of epidermal thickness shown by BD-SA-CBs compared to other groups can be seen. The enhancement of such effect is because of the nanosized range of CBs, special properties of lipids, and alteration in rheology, which enhanced the skin permeation and duration of action [138].

In another study, Ramalheiro et al. prepared rapamycin-loaded CBs (RPM-CBs) using the solvent-evaporation method. Later, these CBs were loaded into rapidly dissolving microneedles (MNs), which were considered beneficial for the long-term treatment of psoriasis. MNs were employed because they can penetrate the stratum corneum and reach the deeper layers of the immunologically rich region of the skin. The reported particle size of RPM-CBs was 221 ± 14 nm with a greater encapsulation efficiency of 94.6 ± 4.0%. The *in vitro* release study revealed the sustained release behavior of RPM from RPM-CBs. It was observed that CBs can diffuse at 310 *μ* depth of the skin, revealing the potential role of CBs in topical skin applications. Generally, natural killer (NK) cells are involved in the etiology of psoriasis. Thus, the biological activity of RPM-CBs was checked on the NK-92 Cl cell line. The NK-92 cells were incubated with different formulations, *viz*., RPM solution, blank CBs, and RPM-CBs for 72 h to know the effect of RPM on cell proliferation. The cells cultivated with RPM-CBs and RPM solution showed a significant reduction in cell proliferation at 82.9 ± 2.5% and 81.9 ± 1.8% at 10 ng/ml and 20 ng/ml, respectively. Moreover, no toxicity was observed with blank CBs, suggesting its nontoxic properties. The overall finding from the study depicted the usefulness of CBs in loading hydrophobic drugs, providing better diffusion, and exhibiting prolonged effects in psoriasis [139].

### 9.2. Skin Cancer

**Figure 6:**
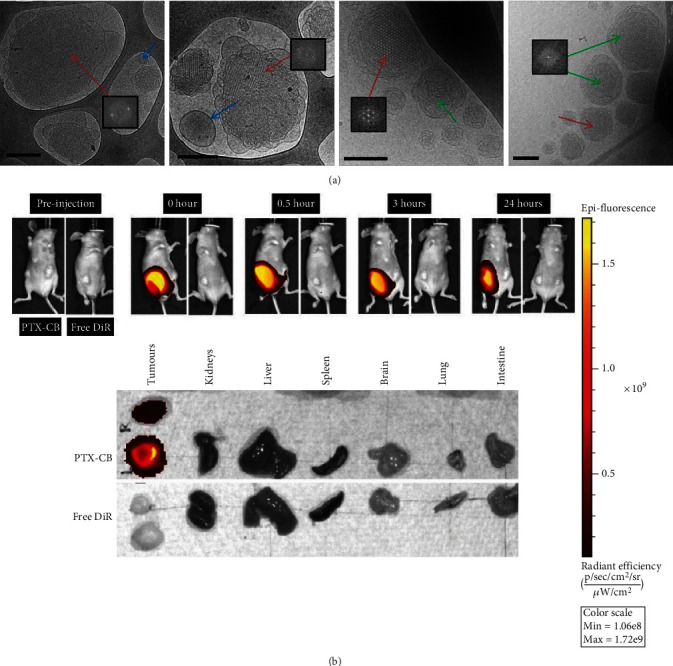
(a) Morphological characterization study by TEM images. (b) *In vivo* biodistribution study of CBs. Reproduced with permission [140]. Copyright © 2022 ACS Publications.

The CBs have the potential to carry hydrophobic chemotherapeutic drugs in their 3D porous structure and, thus, highlight their role as versatile drug delivery carriers in skin cancer. Various studies have been conducted so far to explain the usefulness of CBs in melanoma. Among all, a study by Zhai et al. explained the biological response provided by paclitaxel-loaded CBs (PTX-CBs) in skin cancer using the A431 skin cancer xenograft model. The prepared CBs were spherical, 150-500 nm in size, and had cubic phase symmetry, as shown in Figure 6(a). The *in vitro* toxicity assessment of the CBs was conducted by employing 2D and 3D cultures of human epidermoid carcinoma A431 cell lines. The *in vivo* dose-response study was conducted with different doses of the drugs, in which an effective and safer dose was found to be 150 mg kg^−1^. Through IVIS, authors observed that CBs were more distributed in the tumor region in contrast to other organs when administered via the intraperitoneal route in A431 tumor-bearing mice, as shown in Figure 6(b). The longer residence of the CBs in the tumor region was owed to the slowed diffusion of the nanoparticles. Further, the reduction in tumor volume was found to be two-fold and 0.7-fold with PTX-CBs and free PTX group, respectively. The authors concluded that CBs could act as versatile drug carriers to achieve localized delivery of cytotoxic drugs with enhanced safety in melanoma [140].

In another study, Waheed et al. developed apigenin (AG) loaded CBs for the effective treatment of skin cancer. They observed an enhanced permeation of the AG-CBs, i.e., 170.24 ± 6.81 *μ*g cm^−2^ in contrast to plain AG suspension 70.81 ± 6.2 *μ*g cm^−2^ owing to the presence of GMO and structural similarity of CBs with the skin. The confirmation of such permeation effect was further studied through confocal laser microscopy, and results revealed the six-fold higher penetration of AG-CBs at the lower skin region in contrast to the plain drug (AG-CBs—59.9 *μ*m and AG—10 *μ*m). The findings explain AG-CBs effectively permeated through the stratum corneum as the stratum corneum thickness is 0 to 20 *μ*m. The study also depicted the higher maximum concentration toward skin (*C*_max_) and AUC_0−24_ for CBs, concluding higher accumulation in the desired area of the skin. In addition, the IC_50_ values of AG-CBs and AG-suspension in B16F10 cells were found to be 45.75 ± 0.05 *μ*M and 85.39 ± 1.35 *μ*M, respectively, highlighting the effectiveness of the developed formulation. The obtained results support the significant role played by CBs in achieving effective treatment of skin cancer by penetrating deep into skin layers [141].

### 9.3. Cutaneous Candidiasis

Every year, most fungal infections are caused by *Candida albicans* affecting various parts of the body, i.e., mucosal, skin, and systemic infections. Among all, the skin is affected by cutaneous candidiasis. The mortality rate caused by fungal infections is higher compared to diseases like HIV, malaria, and breast cancer [142]. *Candida albicans* enter the inner layers of tissue via adhesion, invasion, and host cell damage. In addition, the fungus adheres to the epithelial cells in the tissue, followed by hyphal extension with consequent penetration of host cells. This process is controlled by members of adhesion and invasion families [143].

CBs show beneficial effects in candidiasis as described in various studies. The study by Prajapati et al. prepared fluconazole-loaded CBs (FCZ-CBs) using the emulsification method for the treatment of cutaneous candidiasis. The *in vitro* antifungal study was conducted on MTCC 1637 (*Candida albicans*) pathogens by incubating with PBS pH 6.5, FCZ solution, blank CBs, and FCZ-CBs and monitoring the zone of inhibition for 3 to 5 days. It was observed that FCZ-CBs showed more zone of inhibition (22 ± 0.7 mm) compared to other treatment groups, i.e., PBS pH 6.5 (8 ± 0.2 mm), blank CBs (9 ± 0.5 mm) and FCZ solution (15 ± 0.7 mm). Through colony-forming unit (CFU) study, it was found that FCZ-CBs exhibited low CFU (1.27 ± 0.04) with superior activity over the FCZ solution (3.42 ± 0.06) study on rat skin of *Candida albicans*. In addition, the confocal microscopy study specified that FCZ-CBs showed permeation up to 100 *μ*m in the skin, indicating that the formulation efficiently reached the infected layers of the skin. Thus, the authors concluded that FCZ-CBs are a promising delivery system for effectively treating cutaneous candidiasis [144]. Another study by Khalifa et al. described the potential of miconazole nitrate-loaded cubosomal gel (MCN-CBs) for topical delivery against *Candida albicans*. The MCN-CBs were prepared using the emulsification method and then further loaded into a hydrogel. The *ex vivo* study of MCN-CBs gel showed enhanced MCN permeation across the dorsal rabbit skin compared to the commercial cream (Miconaz®). Moreover, MCN-CBs showed significantly higher antifungal activity against *Candida albicans* than commercial cream, revealing a promising delivery system of MCN against fungal infections [26].

### 9.4. Acne

Acne vulgaris occurs when hair follicles plug-in oil and dead skin cells. It is common in teenagers and young adults. Symptoms include the appearance of pus-filled pimples or large, red, and tender bumps [145]. Several intricate elements are combined to produce acne. The amount and size of the sebaceous glands are hereditary, and genetics are assumed to play a significant effect. The hormones, especially androgen (dihydrotestosterone) have an impact on the sebaceous gland. At the time of adolescence, the amount of secretion of hormones released from adrenal glands and gonads is high, and these hormones have a direct effect on the sebaceous glands. When sebum production increases and follicular hyperkeratinization is abnormal, sticky keratinocytes obstruct the pilosebaceous duct and comedones develop. *Propionibacterium acnes*, an anaerobic bacteria, may then colonize the area. Interleukins-12 and 8, as well as TNF, are a few proinflammatory intermediates that *Propionibacterium acnes* uses to promote inflammation [146]. The CBs used for acne are shown to play an important role in reducing adverse effects of drugs like redness, itching, and edema as well as maintaining efficacy by sustaining the release of the drug from the system. Further, enhanced penetration of the drug into the skin layers or pilosebaceous unit is also observed with CBs due to their structure and composition.

To support the role of CBs in acne, Khan et al. formulated erythromycin-loaded CBs (ETM-CBs) by emulsification method, further incorporated into a gel for topical delivery. The CBs were reported as nanosized particles with a high entrapment efficiency of ETM-CBs (95.29 ± 1.32%). The *in vitro* drug release study revealed an initial burst release of ETM from ETM-CBs gel for 24 h in phosphate buffer (pH 6.4). The release of ETM from the water channels in the top layers of the cubic phase is likely responsible for the initial burst release. However, a sustained release of ETM was observed after 24 h, owing to the diffusion pattern of ETM from the inner water channels. They also performed the *in vitro* antibacterial activity of ETM-CBs gel on MTCC 463 (*Saccharomyces cerevisiae*) pathogens and observed that ETM-CBs showed better antibacterial activity over the plain ETM gel, which was due to the enhancement of permeation and retention time of ETM-CBs gel in the agar well plate. These results suggested that ETM-CBs gel has significant antibacterial activity on acne and is suitable for topical application [147].

### 9.5. Alopecia and Vitiligo

Alopecia is a condition that causes hair loss or baldness on the head or other regions of the body where hair is ordinarily found. Based on the causes, there are many varieties of alopecia. Common types of alopecia include alopecia areata, androgenic alopecia, chemotherapy-induced alopecia (CIA), trichotillomania, anagen effluvium, and telogen effluvium [148, 149]. For effective treatment of alopecia areata, Hosny et al. prepared finasteride-oregano oil nano CBs (FS-OG-CBs) using the homogenization technique, which was further loaded into aloe ferox gel. They observed that FS-OG-CBs gel exhibited a sustained and extended release of FS compared to plain FS gel and plain FS suspension. This may be attributed to the nanosize of CBs offering higher surface area for the release of the drug as well as the presence of the drug in the solubilized form in the lipid layer of CBs facilitating the drug dissolution and release. Furthermore, they observed an enhanced permeation rate of FS-OG-CBs gel (1.4 *μ*g cm^−2^ h) on rat skin, which was 4.5-fold higher over the plain suspension (0.31 *μ*g cm^−2^ h). Moreover, they also assessed the antibacterial activity of FS-OG-CBs gel on *Propionibacterium acnes* and found that CBs-based formulation possesses the lowest minimum inhibitory concentration (MIC), i.e., 0.037 *μ*g ml^−1^, compared to plain FS gel (0.211 *μ*g ml^−1^). This may be due to the synergistic effect between FS and OG against *Propionibacterium acnes*. Therefore, this study highlights the potential of FS-OG-CBs gel as a promising strategy for treating alopecia areata [150].

**Figure 7:**
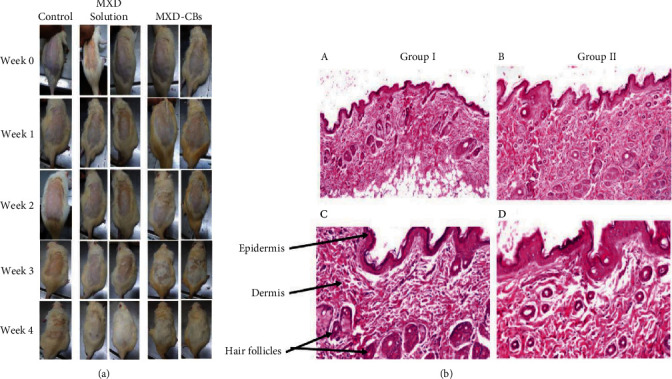
(a) The effect of MXD-CBs when compared to MXD solution and control over one month. (b) The histopathological micrographs of different layers of skin with untreated skin (group I) and MXD-CBs treated group (group II). A, B 16x magnification power and C, D 40x magnification power. Reproduced with permission [99]. Copyright © 2023 Elsevier.

In another study, Makhlouf et al. prepared minoxidil (MXD) CBs (MXD-CBs) by hot melt emulsification technique for hair regrowth. They performed the *in vivo* hair growth study of the prepared MXD-CBs with plain MXD solution on a Wistar rat animal model. The results revealed hair growth after 1 month of topical treatment with MXD-CBs, whereas MXD solution and control groups showed no hair growth (Figure 7(a)). The superior activity of the drug might be due to the unique 3D structure of CBs. Moreover, the lipid present in the CBs functions as a skin adhesive, increasing the therapeutic action of the drug by increasing the retention time of the drug on the skin. The histopathological photomicrographs explained the effectiveness of treatment with MXD-CBs (group II) in the different layers of the skin, and hair follicles were observed when compared to the control (group I), as shown in Figure 7(b) [99].

Vitiligo is a condition in which patchy skin depigmentation can appear in any portion of the body, leading to the loss of pigment cells (melanocytes). Without any appreciable difference between sex, region, and ethnicity, it affects about 1% of the global population [151]. It is thought that vitiligo is an autoimmune disease. This theory is supported by studies showing elevated levels of markers of oxidative stress and autoimmune conditions such as CD8+ cytotoxic T cells, particularly melanocytes as well as by the fact that patients with vitiligo frequently have a personal or family history of other autoimmune conditions. It may also be due to some external factors like chemicals, UV rays, environmental toxins, and chemicals [152]. To support the role of CBs in vitiligo, Sanjana et al. prepared dexamethasone-loaded CBs (DXM-CBs) using the emulsification method, further loaded into carbopol 940 gel, and assessed for the treatment of vitiligo. The prepared DXM-CBs gel had an optimum spreadability (11.6 ± 0.11 g cm sec^−1^) that can be applied effectively as well as leaking can be avoided after administration of it. Moreover, they performed the *in vitro* drug release of DXM from DXM-CBs and DXM-CBs gel using cellophane membrane by the dialysis bag method. Generally, lipophilic drugs require more time to release due to their limited solubility. So, the release of DXM from DXM-CBs was about 88.12% after 12 h in a pH 4.5 buffer. In contrast, 83.58% release of DXM was observed from DXM-CBs gel suggesting that the gel retarded the release due to viscosity. Due to the residential nature of CBs in the skin for an extended period, the authors concluded that CBs gel provides a potential for treating vitiligo topically [153].

## 10. Applications of CBs in Various Ocular Diseases

The bioadhesive nature of CBs efficiently distributes to the surface layers of the eye. The research community has explored CBs for various ocular diseases such as fungal keratitis, glaucoma, conjunctivitis, and uveitis that will be described in the following subsections.

### 10.1. Fungal Keratitis

Fungal keratitis is an infection of the cornea by a fungus. It accounts for 1-45% of infectious keratitis, has a high incidence of approximately 1,000,000 new corneal infections per annum, and a significant risk of blindness. The most common pathogens causing fungal keratitis include *Aspergillus* and *Fusarium*. Fungal infections require growth and survival in the cornea. The temperature in the central part of the human cornea is 32.6 ± 0.70°C, which is suitable for the growth and toxigenicity of *Aspergillus and Fusarium*. Other requirements for fungal infections include penetration to internal tissues despite host defenses, digestion and absorption of nutrients from the host, and resistance to the host immune system [154].

For the effective treatment of fungal keratitis, Said et al. prepared ocular mucoadhesive voriconazole cubosomes (VRZ-CBs) using the hot melt dispersion emulsification technique. Surface modification of VRZ-CBs was achieved with chitosan to enhance the mucoadhesive properties as well as precorneal residence time. The mucoadhesive properties were assessed by mucin to study the behavior of chitosan-coated VRZ-CBs adhesion with the corneal surface in comparison to the aqueous dispersion of mucin. There was an increment in the PS of the chitosan-coated VRZ-CBs dispersion in mucin during its incubation period of 8 h. These results revealed that an excellent mucoadhesive nature was obtained due to the ionic interaction between the surface positive charge of chitosan-coated VRZ-CBs and the negative charge of mucin due to the presence of sialic groups. Moreover, an *in vivo* pharmacokinetic study was performed to know the VRZ concentrations of VRZ suspension and chitosan-coated VRZ-CBs in aqueous humor vs time profiles. After topical instillation, the *C*_max_ and *T*_max_ of VRZ of chitosan-coated VRZ-CBs were 4.44 ± 2.04 ng ml^−1^ and 3.00 ± 0.61 h, respectively, and VRZ suspension were 3.52 ± 1.50 ng ml^−1^ and 2.00 ± 0.40 h, respectively, which depicted a significant increase in the bioavailability of chitosan-coated VRZ-CBs. This was due to an enhanced permeation effect of the lipid monoolein employed in the formulation as well as chitosan, since the positive charge of chitosan could interact with the negative charge of mucin present on the corneal surface. Further, the mean residence time and half-life of chitosan-coated VRZ-CBs were higher compared to VRZ suspension, which may be attributed to higher viscosity and retention of chitosan-coated VRZ-CBs [102].

**Figure 8:**
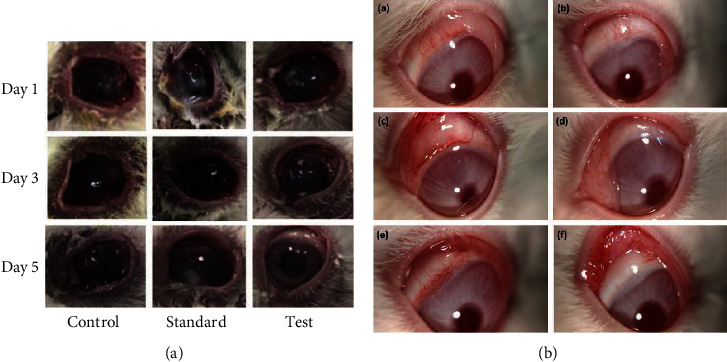
(A) Photographs for the clinical assessment of fungal keratitis in three treatment groups (control-normal saline, standard-KTZ, and test-KTZ-CBs) of rabbits after 1, 3, and 5 days. Reproduced with permission [155]. Copyright © 2023 Elsevier, (B) Photographs of the ocular area on (a, c, e) day 1 and (b, d, f) day 4 after subconjunctival administration of LTP. a, b 0.00125% LTP-CBs, c, d 0.0025% LTP-CBs, and e, f commercial formulation of LTP (Louten®). Reproduced with permission [156]. Copyright © 2023 Elsevier.

In another study, Elfaky et al. prepared ketoconazole-loaded cubic liquid crystalline nanoparticles (KTZ-CBs) for the treatment of ophthalmic mycosis (ocular fungal infections). The authors performed the *in vivo* assessment of ocular irritation and clinical evaluation. As a result, the KTZ-CBs gel showed no evidence of injury or irritation to the conjunctiva which was observed by visual inspection of the rabbit eye. By using a microscope and light slit lamp, the different groups (control-normal saline, standard-KTZ, and test-KTZ-CBs) were examined on the 1^st^, 3^rd^, and 5^th^ day after infection as shown in Figure 8(a). A significant recovery from fungal infection was observed for the KTZ-CBs only on the 5^th^ day. Further, the authors observed that KTZ-CBs gel exhibited a better antifungal effect than the plain KTZ against *Candida albicans*. This finding demonstrates that KTZ-CBs gel effectively treats fungal keratitis when compared to other treatment groups. [155]

As discussed before, *Aspergillus* is one of the most common pathogens in fungal keratitis. Apart from this, *Aspergillus flavus* is found to be the most common etiology of fungal endophthalmitis in India [157]. In a study conducted by Rapalli et al., they prepared ketoconazole-loaded cubosomes (KTZ-CBs) using the hot emulsification technique, which was further loaded into a hydrogel for topical delivery. The authors observed an improved permeation of KTZ from KTZ-CBs gel compared to the marketed product (Ketomac®), which might be due to the occlusive nature of the CBs-based gel. In addition, the antifungal study on MTCC 1403 (*Aspergillus flavus*) depicted a higher zone of inhibition (1.6-folds) for KTZ-CBs compared to the free KTZ. Thus, the authors concluded that KTZ-CBs gel is a promising delivery system for topical drug delivery. This formulation also holds a future scope for delivering BCS class II and IV drugs in topical and ophthalmic applications [53].

### 10.2. Glaucoma

Glaucoma is a series of progressive eye diseases that is characterized by damage to the head portion of the optic nerve due to retinal ganglion cells and their axons. More than 80 million individuals are estimated to be impacted by glaucoma globally and is the primary cause of permanent blindness. Also, it is believed that increased intraocular fluid pressure (IOP) causes retinal ganglion cells to undergo apoptosis, which is swiftly followed by the degeneration of their optic axons. Moreover, the hyperactivity of retinal astrocytes produces the ischemic conditions that are the first symptoms in the pathogenesis of glaucoma [158].

For the treatment of glaucoma, Bessone et al. formulated latanoprost-loaded phytantriol cubosomes (LTP-CBs) using a top-down approach. The authors performed the *in vivo* study on rabbits to determine the efficacy of LTP-CBs with the comparison of the commercial formulation (Louten®). The subconjunctival administration of LTP-CBs in normotensive rabbits showed a 30% decrease in IOP when compared with a 10% reduction of IOP in the commercial formulation (Louten®) within 24 h. This is due to the structure and bioadhesive nature of the CBs. The ocular irritation assay was performed on the anterior segment of the eye and evaluated clinically on day 1 (Figure 8(b), A, C, E) and day 4 (Figure 8(b), B, D, F). The formulation reservoir was visible in the subconjunctival space (Figure 8(b), A, C, E) which was more obvious with the CBs formulation and decreased over time with size, followed by complete fading by day 4 (Figure 8(b), B, D, F). This might be attributed to the slow release of LTP with a prolonged hypotensive effect. There was 15-25% of irritation observed on day 1 which decreased over days. This indicates that the prepared CBs formulation was biocompatible, and irritation was less than 20% and pharmaceutically acceptable for ocular administration [156].

A similar study was performed by Eldeeb et al., where the authors prepared brimonidine tartrate-loaded cubosomes (BRT-CBs) using the melt dispersion emulsification technique. They performed the *ex vivo* study on a rabbit cornea using a Franz diffusion cell. The results revealed a 1.6-fold enhancement in the permeation flux and permeability coefficient of BRT from the BRT-CBs when compared to the marketed product (Alphagan® P). The higher permeability was governed through the exchange of surface lipid between the CBs and epithelial cells of the corneum as well as the structural similarity between the bicontinuous lipid layer of CBs and cells of the corneum which enhances the penetration efficiency of drugs through the corneum. Further, they performed the *in vivo* pharmacodynamic study to know the effect of BRT-CBs and Alphagan® P on IOP. There is a significant decrease in the IOP of BRT-CBs (40.3 ± 3.6%) when compared to Alphagan® P (34.5 ± 3.6%) up to 2 h. The therapeutic effectiveness of the Alphagan® P declined at 5 h and the IOP reverted to normal levels, whereas the BRT-CBs efficacy persisted until the end of the investigation (24 h), resulting in a 6.5 ± 2.9% reduction in IOP. Moreover, there was a 9.1-fold increase in the MRT of BRT-CBs when compared with the Alphagan® P, which was due to the bioadhesive nature of the lipid of CBs and the interaction between the layer of the tear film and CBs [159]. These studies depicted the potential of CBs in the effective treatment of glaucoma.

### 10.3. Conjunctivitis

Conjunctivitis refers to the inflammation of the conjunctiva. There are different etiologies for conjunctivitis, among which certain instances can be brought on by an infection or an allergy. Viral and bacterial conjunctivitis are the two main infectious conjunctivitis among the population. However, allergic conjunctivitis is mainly caused by airborne allergens (pollen, grass, and weeds). Dry eye syndrome, also known as keratoconjunctivitis sicca, is a multifactorial illness linked to several illnesses [160].

In a study conducted by Alharbi et al., they prepared ocular in situ gels loaded with ciprofloxacin cubosomes (CPF-CBs) for the treatment of conjunctivitis and corneal ulcers. They performed the *in vitro* microbiological activity on *Staphylococcus aureus* to evaluate the antimicrobiological activity of the CPF-CBs when compared with the marketed eye drops. There was a more zone of inhibition of CPF-CBs (28 mm) when compared with the marketed eye drops (11 mm). The bicontinuous lipid bilayer of CBs and the membranes of microbial cells share structural similarities, which may have contributed to the ability to fuse membranes and transfer drugs into microbial cells. Additionally, the loading of CPF-CBs into a thermo-gelling base increased the tissue adhesion and hydration, further providing an emollient effect on microbial growth, ensuring close contact with microbial membranes, and promoting efficient drug delivery and increased antimicrobial activity [161].

### 10.4. Uveitis

Uveitis refers to the inflammation of the uvea (iris, ciliary body, and choroid), where any part of the eye can be inflamed. The main pathogenesis of uveitis is not fully known. However, it is believed that uveitis is mediated by a combination of genetic predisposition and cross-immunity to self-antigens or infectious agents [162].

For the treatment of uveitis, Gaballa et al. prepared beclomethasone dipropionate (BMD) cubosomes (BMD-CBs) using the top-down approach. They performed the *ex vivo* transcorneal permeation study on excised bovine corneas using Franz diffusion cells. They observed that there was a 5.7-fold increase in transcorneal permeation of BMD from BMD-CBs when compared with the BMD suspension. This might be due to the employment of GMO lipid in the formulation, as it is an effective transcorneal permeation enhancer. In addition, the surface tension of BMD-CBs was significantly lower than that of the BMD suspension, which might be due to their improved spreading properties, and higher compatibility with the barrier of the precorneal tear film and the lipophilic corneal epithelium. Thus, this lowered surface tension of the optimized BMD-CBs may help in improving the transcorneal penetration of BMD through the corneal epithelium. Moreover, the authors performed the *in vivo* study to determine the residence time in precornea by using sodium fluorescein-loaded CBs. The authors administered sodium fluorescein containing PBS solution (2% w/v), BMD-CBs, and BMD-CBs gel topically to the rabbit eye. They observed that PBS solution was rapidly eliminated approximately after 20 min, while BMD-CBs and BMD-CBs gel remained on the ocular surface (cornea) approximately for a time period of 210 min and 340 min, respectively. The increase in the precorneal residence period of the BMD-CBs gel might be attributed to the cubic structure of CBs that help to retain on the surface of the eye. Thus, this study depicted the potential of CBs in ocular delivery for treating uveitis [163].

## 11. Various Cubosomal Formulations Prepared for Topical Delivery

In addition to CB's role in topical applications for skin and eye diseases, CBs also show prominent topical applications for other diseases, including rheumatoid arthritis, otitis externa, and burns. Various studies have elaborated the topical applications as discussed in Table 6. The investigation explored the CBs role in treating periodontitis (destructive chronic inflammatory disease in the gingival margin). An atorvastatin (ATV) and eugenol (EUG) coloaded cubosomes were formed using a high-shear homogenizer method. The CBs were successfully characterized for particle size, PDI, ZP, morphology, and stability studies. The CBs-loaded *in situ* gel (ATV-CBs-ISG) was formed to acquire the topical applications at the disease site. The gel was found to possess the gelation temperature of 34.00 ± 0.70°C, gelation time of 46 ± 2.82 sec, and viscosity of 7024.5 cps. After successful evaluation of *in situ* gel, the same was assessed for clinical parameters (probing depth (PD), bleeding index (BI), and plaque index (PI)). The study includes 3 groups of patients. Group 1 consists of the nonsurgical scaling (NSC) and debridement (DD) alone (positive control), group 2 received NSC+DD+ATV-CBs-ISG at a dose of 1.2% (treatment control), and group 3 received NSC+DD at a dose of 1.2% ATV-ISG (treatment coarse-drug-loaded gel). The investigation revealed the reduction in PD values from 4.50 ± 0.53 to 1.87 ± 0.64, a reduction in BI values (90%) owing to the anti-inflammatory effect of ATV, and a significant reduction in PI values when treated with ATV-CBs-ISG [164]. The findings from the studies explain the extensive clinical outcomes from CBs in dental needs.

The study by Boge et al. explained the topical delivery of peptide LL-37 for antimicrobial resistance. The high shear homogenization method was adopted using GMO, poloxamer 407, and LL-37 to produce CBs bearing size < 130 nm and PDI < 0.15. The small angle X-ray scattering (SAXS) confirmed the liquid crystalline structure. The *ex vivo* pig skin models explained the antimicrobial resistance and future studies required to be performed to strengthen the mechanism of such [134].

The topical applications of the CBs also find use in the topical treatment of cervical cancer. The study conducted by Victorelli et al. explained the CBs retention at vaginal mucosa. Briefly, curcumin-loaded CBs were formed using biodegradable GMO as lipid and Pluronic F127 as stabilizer. The primitive cubic phase (Im3m) was formed as confirmed by SAXS and cryo-TEM analysis. The particle size, PDI, ZP, and %EE of CBs were found as <200 nm, <0.4, 42.0 ± 1.2 and <86%. The blank CBs, curcumin-loaded CBs, and free curcumin were assessed for *in vitro* cell line studies using HeLa cells for cytotoxicity assay and caspase 3/7 activities. The findings from the studies revealed higher cytotoxicity for curcumin-loaded CBs in a dose-dependent manner than free curcumin; however, blank CBs were found to be nontoxic toward HeLa cells [165]. In addition, curcumin CBs also possess higher caspase 3/7 activities than free curcumin, confirming the effectiveness of formulating CBs for cervical cancer. The future studies using appropriate *in vivo* models can strengthen the CBs' role in such studies.

## 12. Patents on CBs

The potential use of CBs in many domains has been investigated by numerous researchers. Recent patent applications have been filed because of the advancement and success of these investigations, particularly regarding the compositions and methods for preparing CBs for topical delivery. These are listed in Table 7.

## 13. Challenges of CBs

Numerous challenges hamper the progress of CBs in translational topical applications for skin and eye diseases. The biological barriers (eye and skin) and formulation-related obstacles are major issues that require attention during drug carrier development. This section summarizes the challenges associated with topical applications of CBs for skin and eye disease.

### 13.1. Formulation-Related Challenges

The formation of CBs requires suitable lipids and surfactant agents with ideal optimized driving factors, including temperature and concentration of lipids, drugs, and surfactants. Interfacial activity with surrounding water molecules generates serendipitous phase transitions that can lead to structural instability [56]. The change in pH conditions also hampers the physicochemical properties of the lipids, which can lead to phase transitions (psoriasis and skin cancer). In addition, the lower loading capacity for hydrophilic drugs and the large-scale production owing to the higher viscosity of lipids are the challenges associated with the manufacturing of CBs [185].

### 13.2. Biological Challenges

The skin possesses nonviable physical barriers, including the stratum corneum (SC) layer, viable epidermis, dermis, and appendages. The SC layer consists of basal, intermediate, and superficial zones, making the stratum disjunction the main dynamic barrier. Each zone of SC exhibited different thicknesses (4-10 cells thick for basal, 8-12 cells more densely packed for intermediate, and 2-3 cells with intracellular spaces for superficial). These are the SC zones that allow or prevent the penetration of drugs or drug carriers based on their physicochemical properties [186, 187]. The barrier is continuously formed and shed at different times in different skin regions. Generally, it takes three weeks for the back of the hand, two weeks for the back, and one week for the forehead to maintain the actual conditions [188].

Topical applications of CBs persist with multiple ocular barriers, resulting in poor concentrations of drugs at disease sites. These include tear film barrier, nasolacrimal duct drainage, and continuous rapid tear flow [23]. Rapid tear turnover causes dilution of the drug, which governs the low bioavailability of the drugs in aqueous humor owing to the low concentration gradient and diffusion rate [189]. The corneal barrier (external corneal stratified epithelium and stroma) and anterior segment barrier are biological obstacles that can affect the penetration of CBs at different eye segments. The outer eyeball shell, also known as the sclera, is another substantial barrier with limited permeability owing to the thickness of the sclera [190].

Morphological changes in disease conditions (skin and eye) also affect the topical effectiveness of CBs. For example, scaly skin in psoriasis, thickened plaque, and nodules in skin cancer limit penetration and, thus, can reduce treatment efficacy. In addition, sebum production, interindividual responses, and the selection of inappropriate animal models also affect the efficacy of CBs [191]. Evaluating CBs for topical applications, specifically skin, requires a suitable animal model. The fundamental difference between mouse and human skin is that mouse skin is loose and has more hair follicles and a very thin epidermis. Thus, changes in skin condition can alter the final clinical outcomes of topical applications [192].

## 14. Conclusion and Future Perspectives

This review highlights CBs as a promising carrier for topical drug delivery applications. CBs are nanosized liquid crystalline 3D structures formed by the self-assembly of amphiphilic lipids in the presence of a stabilizer. CBs have been recognized as a safe and efficient topical drug delivery system with enhanced retention time. The bioadhesive nature of CBs enables them to be employed in topical as well as mucosal formulations for bioactive delivery. They are useful in enhancing the absorption of poorly water-soluble drugs, safeguarding the underlying substance from enzyme degradation. Concerning the preparation methods for CBs, microfluidics is an emerging approach. Moreover, the structural resemblance between the cubic phase of CBs and the skin stratum corneum aids in achieving more efficient CBs penetration. The biomembrane-type framework of the cubic phase improves physical adherence to the eye and skin, thus enhancing the retention of CBs in the epidermis and dermis layers of the skin and different layers of the eye.

However, with their positive aspects, CBs possess some challenges, e.g., an increase in viscosity is observed during large-scale production batches and difficulty in loading hydrophilic drugs. The rapid release rate of hydrophilic drugs in an aqueous environment should be addressed. The production of CBs is constrained by the absence of regulatory guidance. To commercialize CBs formulations, GMP processes must be more rigorous, including methodology and quality evaluation protocols.

Nevertheless, the burst release of hydrophilic drugs can be altered by the surface modification of CBs. The surface modifications of CBs by suitable ligands (polymer, proteins, aptamers, and peptides) can also confer disease specificity. We believe that pH-responsive and magnetic field-triggered CBs can be engineered in the future to achieve more precise disease specificity via topical route. Such stimulus-sensitive systems with programed release can also be designed for precisely controlled drug release.

Despite the potential of CBs as nanocarriers for topical drug delivery systems, there is limited literature available concerning their fate after *in vivo* administration. In addition, *in vitro* studies have been conducted on confined cell lines/models. Most studies in the literature to date have focused on the preparation, optimization, and basic characterization of CBs. To gather further information on CBs, research on future investigations should be conducted by combining *in vitro* and *in vivo* studies and should provide insights into their toxicity and drug-release properties. Furthermore, upon extensive exploration, CBs could become a widely used drug delivery tool in the research community as new lipid nanocarriers, expanding their clinical applications to cosmetics, biomacromolecular delivery, and immunoassays. Considering all this, we expect an upsurge of research on CBs in the literature within the upcoming years.

## Figures and Tables

**Table 1:** Conventional treatment options and their challenges.

Sr. no.	Disease	Conventional treatment options	Marketed formulations	Challenges	References
1.	Acne vulgaris	Topical gel	Adapalene 0.1% and benzoyl peroxide 2.5% (EPIDUO® gel, Galderma Laboratories), adapalene 0.1% (Differin® gel, Galderma Laboratories)	Washing out of the gel *via* perspiration, less penetration, low efficacy, and tolerability led to poor bioavailability	[41]
2.	Psoriasis	Topical ointment	Tacrolimus 0.1% (Protopic ointment Astellas®), calcipotriol, and betamethasone (Dovobet® ointment, LEO Pharma)	Nonuniform distribution, less absorption, and less cutaneous localization effect led to less effectiveness	[42]
3.	Alopecia	Topical solution	Minoxidil 2% (MINTOP™ solution, Dr. Reddy's Laboratories)	Scalp dryness, burning, redness, and allergic contact dermatitis. These effects may be due to the employment of organic vehicles (alcohols, DMSO, and DMF) as solvents in the formulations	[29, 43]
4.	Fungal infections	Topical cream	Miconazole nitrate (Daktarin® T cream, Johnson and Johnson), and bifonazole (Canespor® cream, Kern Pharma S.L.)	Low skin penetration, skin burning, low absorption, and less stability	[20]
5.	Vitiligo	Topical cream and gel	Clobetasol propionate 0.05% (Temovate cream, Taro Pharmaceuticals), desoximetasone 0.25% (Topicort cream and gel, Taro Pharma Inds. Ltd.), and 0.05% augmented betamethasone dipropionate (diprolene)	Systemic absorption, development of cutaneous atrophy, and telangiectasia are some adverse events associated with conventional topical corticosteroids	[44, 45]
6.	Glaucoma	Topical gel	Timolol maleate (Timoptic-XE, Merck & Co.) and Nyogel (Novartis AG)	Blurred vision	[46, 47]
7.	Fungal keratitis	Ophthalmic suspension	Natamycin 5% (Natacyn®, Alcon)	Vision changes, eye redness, or irritation	[4, 48]
8.	Conjunctivitis	Topical solution	Azithromycin ophthalmic solution 1% w/w (AzaSite™, Inspire Pharmaceuticals, Inc.)	Eye irritation and headache	[49, 50]
9.	Skin cancer	Topical cream	5-Fluorouracil 0.5% (Carac® cream, Valeant Pharma North)	Poor penetration through the skin and tumor and low drug retention at the disease site	[51, 52]

**Table 2:** Brief description of the lipids and stabilizers used in the preparation of CBs.

Name	Structure	Brief description	References
Amphiphilic lipid			
GMO	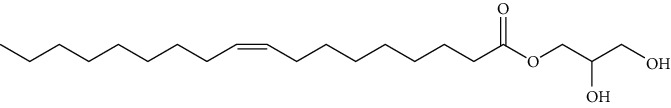	GMO consists of a glyceride of oleic acid and other fatty acids, primarily monooleate, with carbon chain lengths between 12 and 22, forming a cubic structure. GMO is a biodegradable lipid owing to the presence of an ester bond and thus classified as generally regarded as a safe (GRAS) excipient.	[69, 70]
Phytantriol	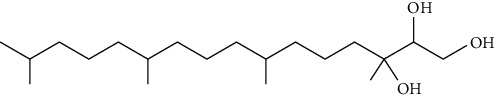	It consists of polyhydroxy alcohol with three hydroxyl groups on one side of the molecule and a long hydrocarbon chain on the other side of the molecule. Phytantriol is stable under aqueous conditions because it does not contain an ester bond in the structure.	[66, 71]
Monoelaidin	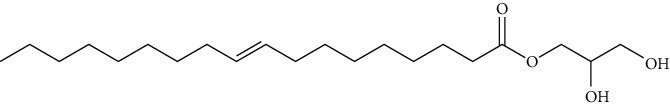	At position 9 and position 10 of its straight acyl chain, monoelaidin possesses a transdouble-bond (C18:19). The self-assembled monoelaidin/water system goes through lamellar to nonlamellar transitions, making it ideally suited to serve as a model system simulating the many stages of membrane fusion that take place in actual cells.	[72]
Stabilizer			
Pluronic F127	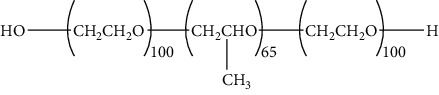	Pluronics are a kind of self-assembling triblock copolymers made up of PEO (polyethylene oxide) and PPO (polypropylene oxide) in the arrangement PEO-PPO-PEO, where PPO contributes to the hydrophobic, and PEO contributes to the hydrophilic nature. The primary function of the stabilizer is to provide an electrostatic or steric barrier that inhibits the interaction between the particles. The hydrophobic PPO block is assumed to adsorb or integrate into the particle's surface and stabilize it in PEO-PPO-PEO. At the same time, the PEO, a hydrophilic component, stretches into the aqueous environment to offer an electrostatic or steric barrier.	[73, 74]
Poloxamine 908	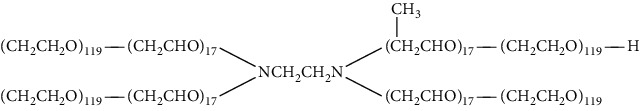	Poloxamine 908, sometimes called Tetronic 908, is a tetrafunctional PEG-PPO ethylenediamine block copolymer. It is also used to stabilize cubosomal dispersions.	[75]

**Table 3:** Advantages and disadvantages of CBs fabrication techniques.

Techniques	Advantages	Disadvantages
Top-down approach (1) High-pressure homogenization (2) High-shear homogenization (3) Probe sonication	It provides a lower particle size with a lower polydispersity index (PDI) [116].	Chances of thermal degradation of lipids and drugs and denaturation of amino acid-containing molecules [69].
Bottom-up approach (1) Sonication (2) Vortex (3) Stirring	It requires less energy input and is useful for thermosensitive agents [69].	Certain drawbacks associated with hydrotropes include the risk of allergic response and inflammation [68].
Spray-drying approach	This method is affordable and typically simple to scale up [117].	The problem associated with the spray drying technique is the formation of a cubic phase immediately after the hydration of lipids [117].
Microfluidics approach	This approach can be employed to prepare stable CBs and load siRNA. Scale-up is possible, and a controlled particle size can be obtained [114].	Specialized equipment is required [114].

**Table 4:** Examples of CBs produced using different manufacturing techniques.

Sr. no.	Technique	Lipid and bioactive	Process parameters	Responses	References
1	High-shear homogenization	Glyceryl monooleate-flurbiprofen	Temperature 80°C with speed 11000 rpm for 3 min	Particle size 130-150 nm, PDI < 0.132, ZP -15 mV, and %EE of >98%	[118]
2	High-pressure homogenizer	Phytantriol- amphotericin B	1200 bar pressure with 9 cycles	Particle size 266.0 ± 3.2, PDI 0.12 ± 0.02, and %EE of 88.5 ± 0.6	[119]
3	High-pressure homogenizer and spray drying	GMO-vinpocetine	Pressure 1200 bar for 5 cycles and spray dryer 40 m3/h, spray flow rate of 600 L/h, and pump setting of 2.72 mL/min	Particle size 246.5 ± 45.7 nm, ZP 30 mV, and %EE 96.4	[120]
4	Hot emulsification and high-pressure homogenization	GMO- sumatriptan succinate	70°C for emulsification and 10 cycles at 700 bar pressure for HPH	Particle size 76-147 nm, ZP + 11 to +15 mV, and %EE of 78-81%	[121]
5	Hot emulsification and high-speed homogenization	GMO-celecoxib	Water bath temperature 70°C and homogenization at 10,000 rpm for 5 min	Particle size ranges from 130 ± 2.93 nm to 191.40 ± 2.356 nm and ZP -23.10 ± 1.68 mV to −37.40 ± 0.99 mV	[122]
6	Hot emulsification and ultrasonication	GMO-Gemifloxacin mesylate	The melting temperature for lipid was 75°C, the rate of addition into the aqueous phase was 1200 rpm, and ultrasonication conditions were 70% amplitude (1 pulse on and 1 pulse off)	Particle size 115.2 ± 2.92 nm, PDI 0.372 ± 0.022, and %EE of 96.62 ± 2.35	[123]
7	Ultrasonication	GMO-dapsone	Probe sonication for 15 min at 15 s on and 5 s off	Particle size 39.4 ± 3.6 nm, PDI 3.53 ± 0.03, ZP -2.1 ± 0.7, and %EE of 88.8 ± 3.7	[124]
8	Probe sonication	Hyaluronic acid-GMO-copper acetylacetonate	Sonication for 30 min in 1 s pulse on 1 s off at 80% amplitude in ice bath	Particle size 125-152 nm, PDI 0.131-0.159, and ZP 40.8 mV	[125]
9	Spray drying	Chitosan-GMO-paliperidone palmitate	Inlet temperature was 130°C, spray gas flow was 357 L/h, and flow rate was 3 mL/min	Particle size 249.8 ± 15.9, PDI 0.353 ± 0.014, ZP −9.0 ± 0.7, and %EE 99.7 ± 0.1%	[126]
10	Spray drying	GMO-erlotinib	Inlet temperature 65-95°C	Particle size 453.40 ± 14.65 nm, PDI 0.502 ± 0.03, ZP −28.6 ± 1.38 mV, and %EE 96.2 ± 3.98%	[127, 128]
11	Microfluidics	GMO, Phytantriol, and Tocopherol lipids and acetate-curcumin and 5(6)-carboxyfluorescein	Flow rate of organic and aqueous phases was 100 *μ*L/min, and the flow rate of the central channel was 20 *μ*L/min. The total flow rate was 480 *μ*L/min.	Particle size 130-300 nm, PDI 0.07-0.14.	[129]
12	Microfluidics	GMO-siRNA	Reynold number 2-500, total flow rate 0.02 mL-4 mL/min	Particles size 201 nm, PDI 0.04, and gene knockdown efficiency of 73.6%	[114]

**Table 5:** Various techniques are used for the characterization of CBs.

Sr. no.	Evaluation parameters	Techniques	Importance	References
1.	Hydrodynamic particle size, PDI, and zeta potential	Dynamic light scattering (DLS) and electrokinetics	Determine the effect of size and surface charge on dissolution, skin permeation, and adhesiveness.	[130]
2.	Thermograms	Differential scanning calorimetry (DSC)	Determine the phase transition of materials.	[112, 131]
3.	Shape	Scanning Electron Microscopy (SEM)	Determine the surface morphologies of CBs.	[132]
4.	Shape	Cryo-Transmission electron microscopy (TEM)	Determine the internal morphologies of CBs.	[133]
5.	Identification of phases	Small-angle X-ray scattering (SAXS)	Determine the structural configuration of the particles.	[134]
6.	Crystallinity	X-ray diffraction (XRD) and DSC	To characterize the phase identification of crystalline materials.	[134]

**Table 6:** Recent advances in the use of CBs in topical applications for treating diseases other than skin and ocular diseases.

Sr. no.	Formulation	Lipid	Stabilizer	Method of preparation	Applications	Reference
1.	Dapsone-loaded CBs (DS-CBs) for enhanced permeation across the skin	GMO	Poloxamer 407	Ultrasonication	The *ex vivo* permeation study revealed higher permeation of DS from DS-CBs (71.28 ± 4.65 *μ*m/cm^2^/h) compared to the marketed DS (55.28 ± 2.13 *μ*m/cm^2^/h) across pig ear skin, i.e., 1.3-fold penetration of DS over time.	[124]
2.	Methoxetrate-loaded CBs (MT-CBs) for topical treatment of rheumatoid arthritis	Cetyl palmitate	Poloxamer 188	Lipid emulsification, followed by high-pressure homogenization	The *ex vivo* permeation of MT-CBs across rat skin resulted in sustained permeation up to 12 h. Also, the retention of MT-CBs after staining was observed by an optical microscope. No skin irritation of MT-CBs was observed. Further, the *in vivo* antiarthritic study revealed that MT-CBs possess a greater analgesic effect on rat over diclofenac gel.	[166]
3.	Norfloxacin-loaded nano-CBs (NO-CBs) for ototopical treatment of otitis externa	GMO	Poloxamer 108	Emulsification	The *ex vivo* permeation study depicted that NO-CBs could permeate the rabbit ear more effectively when compared to NO suspension. Moreover, the *in vivo* skin deposition study across rabbit ear skin resulted in higher NO deposition than the NO suspension. The *in vivo* histopathological investigation of the rabbit ear skin layers also confirmed the safety of the applied NO-CBs on the skin of the rabbit ear, where no irritation was seen.	[167]
4.	Silver sulfadiazine-based CBs (SS-CBs) hydrogels for topical treatment of burns	Monoolein	Poloxamer 407	Emulsification	The *in vivo* histopathological examination of the rat skin revealed that SS-CBs were more effective when compared to the marketed cream (Dermazin®) for treating burns.	[168]
5.	Curcumin-based CBs (CU-CBs) hydrogel for antibacterial activity	GMO	Poloxamer 407	Emulsification, followed by homogenization	The zone of inhibition of CU-CBs was significantly higher than the pure CU, resulting in enhanced antibacterial activity.	[169]
6.	Ketoprofen-based cubogel (KP-CBs) for topical sustained delivery in arthritis	GMO	Poloxamer 407	Emulsification, followed by homogenization	Sustained release of KP from KP-CBs compared to the plain KP gel revealing the potential application of KP-CBs in arthritis.	[170]
7.	Dexamethasone acetate-based cubogel (DXM-CBs) for treating skin inflammation	GMO	Poloxamer 407	Hot melt ultrasonication	Higher penetration and intradermal retention rates of DXM-CBs were observed when compared to the commercial DMX cream, revealing the potential of DXM-CBs for dermatitis by percutaneous drug delivery.	[28]

**Table 7:** Recent patents on CBs.

Sr. no.	Patent no.	Year of filing	Filing country	Inventor	Description of the patent	Status	Reference
1.	KR102458630	2021	South Korea	Ji Hong Geun, Park Young Ah, Kang Yu Jin, Son Seung Yeon, and Lee Dong Won	Cosmetic composition containing retinal-loaded cubosome and covalently bonded organic framework for transdermal delivery	Granted	[171]
2.	KR102307587B1	2019	South Korea	Kim Jin-cheol and Park Seok-ho	Monoolein CBs exhibiting a pH-responsive property were prepared. The active ingredient used was Bambusae caulis in Teaniam (BCT). In the aqueous channel of the cubic phase, albumin and pectin structures were modified (using hydrophobic aliphatic group). This modification controlled the release of BCT at specific pH or temperature	Granted	[172]
3.	KR102310044B1	2019	South Korea	Chang-soon, Kim Hong-rae, Kim Da-young, and Choi Won-seok	Lutein encapsulated in CBs (LT-CBs) exhibited easy penetration through the skin. The encapsulated lutein was derived from PB17 (Auxenochlorella protothecoides, Chlorella minutissima), that was cultured using the phycoil signal process (PSP) method and separated in a supercritical manner. LT-CBs were made up of monoolein and fall in the range of 100-200 nm by showing 30-40% permeation across artificial skin membrane. It showed excellent skin permeation, antioxidant, whitening, and antiwrinkle effects, with no skin irritation	Granted	[173]
4.	KR102105256B1	2018	South Korea	Kim Ji-hyeon, Seo Eun-kyung, Nam Seung-ok, and Kim Yu-jun	The prepared cubic phase lipid nanostructures encapsulating the active ingredient Phyllostachyos caulis in Taeniam extract (PCT) showed an improved moisturizing and antioxidant effects by evading irritation to the sensitive skin. The preparation can be used in many applications like skin softeners, face masks, eyeliner, and creams	Granted	[174]
5.	KR102013804B1	2016	South Korea	Park Sang-hyeon and Lee Kwang-sik	CBs were made up of monoacylglycerol (70–80%), polymer (5–10%), antioxidants (0.1–1%) like BHT, tocopherol, and 20% water. Ceramide (0.1–0.5%) and phytosphingosine were used as the main ingredients. The prepared CBs were 10 nm to 1 *μ*m in size. It showed excellent skin moisturizing effect and barrier improvement of skin	Granted	[175]
6.	CN106619573A	2016	China	Wu Chuanbin Yang Li, Li Yanrong Pan Xin Huang Ying, Chen Hangping	Glyceryl monooleate (GMO) (1-18 part), poloxamer 188 (0.2-0.3 part), EL-60 used in the preparation of timolol maleate cubic liquid crystal nano eyedrop to increase the corneal osmosis, reduced intraocular pressure, increment in retention time and sustained release	Granted	[176]
7.	KR1020210085164	2019	South Korea	Kim Jin Chul and Park Seok Ho	pH-responsive cubosome containing active constituent (BCT protein) in which the release rate can be easily controlled according to pH due to the use of GMO and pectin or GMO and albumin.	Granted	[177]
8.	KR1020180032842	2018	South Korea	Park Sang Hyun, Lee Kwang Sik and Lee Kun Kook	Cosmetic composition containing phytosphingosine and ceramide-loaded cubosome for improving the solubility of phytosphingosine and ceramide, rate of penetration, and persistence	Granted	[178]
9.	US8414914B2	2012	USA	Philip James Bromley and Lee Nickols Huang	The compositions and methods for formulating emulsions further loaded to a micelle, liposome, and cubosome were described. The formulation was intended for administration to the gastrointestinal, oral, and nasal mucosa	Granted	[179]
10.	US7959935B2	2005	USA	Steven B. Hoath, William L. Pickens, Martha O. Visscher, Anyarporn Tansirikongkol, and Richard Randall Wickett	A simulated vernix composed of hydrated synthetic cells in a lipid matrix or water-in-oil emulsified particles or cubosomes/water with 30% protein and 5-30% lipid that provide hydration, cleansing, and other properties	Granted	[180]
11.	KR1020050011153	2003	South Korea	Choi Gun Ho, Lee Seung Hwa, and Lee Shinehee	Cubosomes containing the active ingredient (Trifolium repens L. extract) for antiaging activity resulted in enhanced elasticity and moisturizing effect of the skin, leading to minimal moisture loss of keratin	Granted	[181]
12.	IN202321011171	2023	India	Dr. Upendra Chandrakant Galgatte, Ms. Shruti Ramesh Kolsure, and Dr. Praveen Digambar Chaudhari	Topiramate-loaded cubosome for nasal spray offered the potential to increase the drug's bioavailability as well as residence time and reduced drug dosage frequency	Pending	[182]
13.	IN202141018014	2021	India	Mr. Bhaskar Kurangi, Dr. Sunil Jalalpure, and Mr. Satveer Jagwani	Transdermal cubosomal gel containing resveratrol and piperine showed potential anticancer activity for managing melanoma. The composition of cubosomal gel includes monoolein (3-5% w/v), pluronic F-127 (1-1.5% w/v), resveratrol and piperine (0.1% w/v each), and carbopol 934 (1% w/v)	Pending	[183]
14.	WO2005077336A1	2005	South Korea	Hesson Chung, Seo-Young Jeong, Ick-Chan Kwon, Soo-Yeon Lee, and Kyung-Ho Roh	The formulation and composition of sterically stabilized emulsion and cubosome were described. The sterically stabilized cubosomes contain phytantriol (0.1-30 weight %), drug (0.1-10 weight %), oils (0.1-6 weight %), biocompatible hydrophilic polymer (0.01-5 weight %), and water or aqueous solution	Pending	[184]

## Abbreviations

%EE: Percentage entrapment efficiency
PDMA-b-PNIPAM: Poly (N, N-dimethylacrylamide)-block poly (N-isopropylacrylamide)
2D: Two-dimensional
3D: Three-dimensional
AG: Apigenin
ATV: Atorvastatin
BCT: Bambusae caulis in Teaniam
BD: Betamethasone
BHT: Butylated hydroxytoluene
BI: Bleeding index
BMD: Beclomethasone
BPS: Phytosterols
BRT: Brimonidine tartrate
CH: Chitosan
CIA: Chemotherapy-induced alopecia
CPF: Ciprofloxacin
CPP: Critical packing parameter
CU: Curcumin
CBs: Cubosomes
CYP450: Cytochrome P450
DD: Debridement
DLGL: Sodium dilauramidoglutamide lysine
DLS: Dynamic light scattering
DMF: Dimethyl formamide
DMSO: Dimethyl sulfoxide
DNA: Deoxyribonucleic acid
DPPS: Dipalmitoyl phosphatidylserine
DS: Dapsone
DSC: Differential scanning calorimetry
DXM: Dexamethasone
ETM: Erythromycin
EUG: Eugenol
FCZ: Fluconazole
Fd3m: Discontinuous type
FS: Finasteride
GBD: Global burden of disease
GMO: Glyceryl monooleate
GRAS: Generally regarded as safe
HII: Hexagonal phase
HIV: Human immunodeficiency virus
Ia3d, CG: Gyroid type
III: Micellar cubic phase
Im3m, CP: Primitive type
IMQ: Imiquimod
IOP: Intraocular pressure
ISG: *In situ* gel
IVIS: *In vivo* imaging system
KP: Ketoprofen
KTZ: Ketoconazole
LII: Emulsified microemulsion
LT: Lutein
LTP: Latanoprost
MNs: Microneedles
MPL: Monophosphoryl lipid A
MT: Methotrexate
MTCC: Microbial-type culture collection
MXD: Minoxidil
NK: Natural killer
NO: Norfloxacin
NSC: Nonsurgical scaling
OG: Oregano oil
PASI: Psoriasis area and severity index
PCT: Phyllostachyos caulis in the Taeniam extract
PD: Probing depth
PDI: Polydispersity index
PEG: Polyethylene glycol
PEO: Polyethylene oxide
PHY: Phytantriol
PI: Plaque index
PMA: Poly (methyl acrylate)
Pn3m, CD: Double-diamond type
PPEGMA: Poly (poly (ethylene glycol) methyl ether acrylate)
PPO: Polypropylene oxide
PS: Particle size
PSP: Phycoil signaling process
PTX: Paclitaxel
QII: Inverse bicontinuous cubic phase
Quil A: Extract of the bark of the *Quillaja saponaria* tree
Re: Reynolds number
rpm: Revolutions per minute
RPM: Rapamycin
SA: Salicylic acid
SAXS: Small-angle X-ray scattering
SC: Stratum corneum
SCMC: Sodium carboxymethylcellulose
SEM: Scanning electron microscopy (SEM)
SHM: Staggered herringbone mixer
siRNA: Small interfering ribonucleic acid
SS: Silver sulfadiazine
TDDS: Topical drug delivery systems
TEM: Transmission electron microscopy
TNF: Tumor necrosis factor
TPGS: D-*α*-tocopheryl poly (ethylene oxide) 1000 succinate
VitE-A: Vitamin E acetate
VRZ: Voriconazole
WHO: World Health Organization
XRD: X-ray diffraction
ZP: Zeta potential.
